# Separation of *cis*- and *trans*-Asarone from *Acorus tatarinowii* by Preparative Gas Chromatography

**DOI:** 10.1155/2012/402081

**Published:** 2012-01-12

**Authors:** H. L. Zuo, F. Q. Yang, X. M. Zhang, Z. N. Xia

**Affiliations:** College of Chemistry and Chemical Engineering, Chongqing University, Chongqing 400030, China

## Abstract

A preparative gas chromatography (pGC) method was developed for the separation of isomers (*cis*- and *trans*-asarone) from essential oil of *Acorus tatarinowii*. The oil was primarily fractionated by silica gel chromatography using different ratios of petroleum ether and ethyl acetate as gradient elution solvents. And then the fraction that contains mixture of the isomers was further separated by pGC. The compounds were separated on a stainless steel column packed with 10% OV-101 (3 m × 6 mm, i.d.), and then the effluent was split into two gas flows. One percent of the effluent passed to the flame ionization detector (FID) for detection and the remaining 99% was directed to the fraction collector. Two isomers were collected after 90 single injections (5 uL) with the yield of 178 mg and 82 mg, respectively. Furthermore, the structures of the obtained compounds were identified as *cis*- and *trans*-asarone by ^1^H- and ^13^C-NMR spectra, respectively.

## 1. Introduction

Isomers are quite common in the essential oils from natural plants. *Cis*-/*trans*- or *β*-/*α*-asarones are the main active components in *Acorus tatarinowii* Schott, a traditional Chinese herbal medicine commonly used for improvement of learning and memory [1]. Asarones are known carcinogenic compounds [2, 3]. Actually, the neuroprotective effects of *α*-asarone [4, 5] and *β*-asarone [5–11] have been intensively studied, while the *α*-asarone was found to exhibit more potent effects than the *β*-asarone [5]. Furthermore, *α*-asarone was reported to reduce the incidence and duration of tonic seizures induced by maximal electroshock [12] and by pentylenetetrazole [13]. Further study indicated that activities of *α*-asarone in various animal seizure models [14] and against noise-stress effect [15] might be essentially accounted for by antioxidant properties. In addition, the hypolipidaemic [16–20] and radioprotective effects [21] of *α*-asarone were also been reported. Besides, *β*-asarone, which has toxic and sterilizing effects, shows potential for stored-product pest control [22].

On the other hand, the isomers usually have the similar mass spectra, which will make the accurate identification of those compounds impossible only based on the mass data. For example, furanodienone could be completely transformed into curzerenone during GC-MS analysis of essential oils from *Curcuma* rhizome [23]. And the mass spectra of furanodienone and curzerenone were very similar, which induced the wrong identification of furanodienone as curzerenone without reference compound [24]. To date, HPLC [25–28] and GC-MS [29–32] are two most frequently used techniques for the analysis of asarone. Furthermore, asarone isomers also show similar mass spectra [33], which make the identification of those compounds difficult when the reference compounds were absence. Therefore, the separation and purification of chemical compounds for those isomers are important for the analytical purpose. However, conventional silica gel chromatography is often insufficient to resolve closely related substances and isomers [33]. Gas chromatography provides better separation for many organic compounds. Therefore, preparative gas chromatography (pGC) should be a promising alternative or supplement for fractionation of isomers from the essential oils. As the continuous study of our previous report [34], the pGC was applied for the isolation of *cis- *and *trans-*asarone from the essential oil of *Acorus tatarinowii*.

## 2. Materials and Methods

### 2.1. Materials

Essential oil of *Acorus tatarinowii* was purchased from Jiangxi ji'an FuDa Nature Medical Oil Factory (Jiangxi, China). Silica gel (100~200 mesh and 200~300 mesh) for column chromatography and silica gel (GF_254_) for thin layer chromatography (TLC) were purchased from Branch of Qingdao Haiyang Chemical Plant (Branch of Qingdao Haiyang Chemical Co., Ltd. Shandong, China). Petroleum ether (PE) and Ethyl acetate (EA) were of analytical grade (Chuandong Chemical Co., Ltd. Chongqing, China). The voucher specimen of *Acorus tatarinowii* oil was deposited at the Department of Pharmaceutics, College of Chemistry and Chemical Engineering, Chongqing University, Chongqing, China.

### 2.2. Sample Preparation-Silica Gel Chromatography

In brief, 53.6 g essential oil of *Acorus tatarinowii* was mixed with 100~200 mesh silica gel (the ratio was about 1 : 1.2), and then the mixed sample was subjected onto a column (60 cm × 6.0 cm, o.d.) packed with 800 g 200~300 mesh silica gel and washed by different ratios of PE and EA as gradient elution solvents. The higher polarity part of the fractions eluted by PE : EA = 20 : 1 was collected and we repeated the separation by silica gel chromatography until the pure mixture of the asarone isomers (a single claret-colored spot detected by TLC) was obtained. Then the target effluent was collected (about 2 g of the mixture of asarone isomers were obtained) and condensed before injected into pGC system.

### 2.3. pGC System

The pGC system was modified based on an SC-2000 GC instrument (Chuanyi Analyzer Co. Ltd. Chongqing, China) [34]. It is equipped with a stainless steel column packed with 10% OV-101 (3 m × 6 mm, i.d.), a flame ionization detector (FID), a special effluent splitter with minimum dead volume, and a home-made preparative fraction collector. The data was collected and analyzed on a HW-2000 Chromatographic Workstation (Nanjing Qianpu Software Co. Ltd., China).

High-purity nitrogen (N_2_) was used as carrier gas at a flow rate of 25 mL/min. The inlet and FID temperature were 230°C and 250°C, respectively. The column temperature was isocratic at 220°C. The effluent was splitted into two flows, one (1%) towards the FID and the other (99%) to the fraction collector using a special gas effluent splitter. Two restrictor valves were used to control the split flow. In order to supply sufficient gas flow for the FID detection, a supplementary gas (N_2_, 10 mL/min) was added before arrived at the detector. Volumes of 5 *μ*L asarone isomers mixture were injected. After being separated by the column, the fractions were collected in 2 mL traps filled with ethyl acetate. The trapping time and peak retention time were synchro. The isolated fractions were analyzed by capillary GC and GC-MS.

### 2.4. GC-FID and GC-MS Analysis

The purity identification was performed on an SC-6000 GC instrument (Chuanyi Analyzer Co. Ltd. Chongqing, China). Compounds were separated on an AC-5 fused silica capillary column (30 m × 0.32 mm, 0.25 *μ*m, SGE). The inlet and FID temperature were 250°C and 270°C, respectively. Injection volume was 0.1 *μ*L. Carrier gas was N_2_. The column temperature was maintained at 105°C for 10 min, and ramped at 10°C min^−1^ to 150°C, kept at 150°C for 1 min, then ramped at 5°C min^−1^ to 180°C, eventually to 230°C with the speed of 10°C min^−1^, and kept at 230°C for 2 min.

GC-MS was performed on a Trace GC Ultra gas chromatography instrument coupled to a DSQ II mass spectrometer and an Xcalibur Version 2.0.7 software (Thermo Fisher Scientific, Boston, MA, USA). Compounds were separated on an HP-5MS (30 m × 0.25 mm, i.d.) capillary column coated with 0.25 *μ*m film 5% phenyl methyl siloxane. The temperature of the column was maintained at 80°C for 2 min and then ramped at 8°C min^−1^ to 220°C. Split injection with a split ratio of 1 : 19 and high-purity helium was used as carrier gas with the flow rate of 0.9 mL/min. The spectrometer was operated in electron-impact (EI) mode, the scan range was 10–900 amu, the ionization energy was 70 eV, and the scan rate was 0.34 s/scan. The injection temperature and ionization source temperature were 300 and 230°C, respectively.

## 3. Results and Discussion

### 3.1. Isolation of Asarone Isomers by pGC

**Figure 1:**
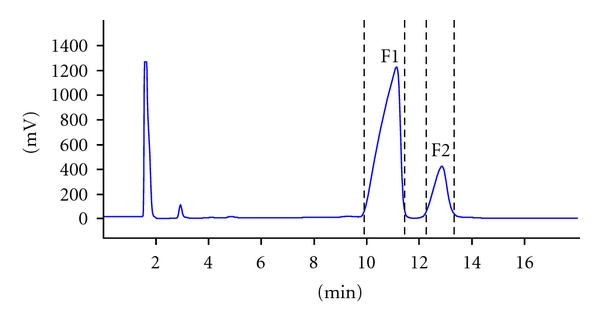
pGC-FID chromatogram for asarone isomers.

The GC chromatogram of asarone isomers mixture recorded by pGC with FID detection is given in Figure 1. It was used as a basis for the collection of two fractions (F1 and F2) that were analyzed by the capillary GC and GC-MS system for an evaluation of resolution and yields of the pGC. After 90 injections, it was resulting in amounts of 178 mg (F1) and 82 mg (F2) for the compounds in the respective traps, respectively.

### 3.2. Identification and Yield of Collected Fractions

**Figure 2:**
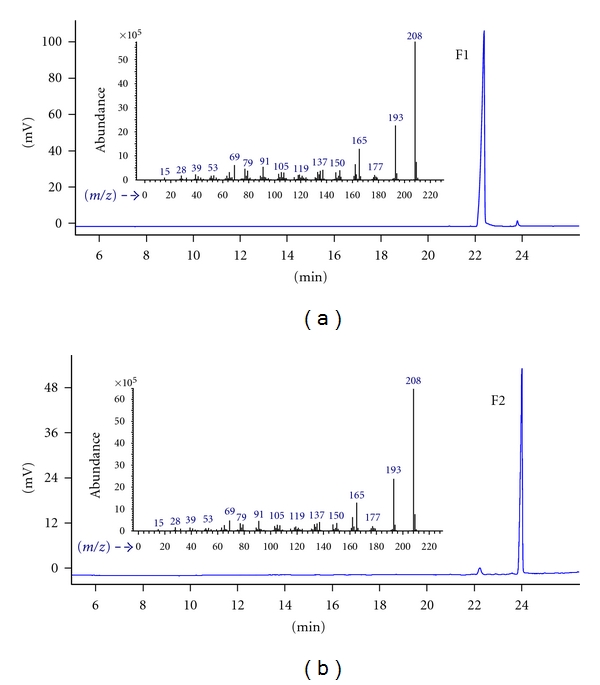
Capillary GC-FID chromatogram and MS spectra for the collected fractions (F1 and F2).

**Figure 3:**
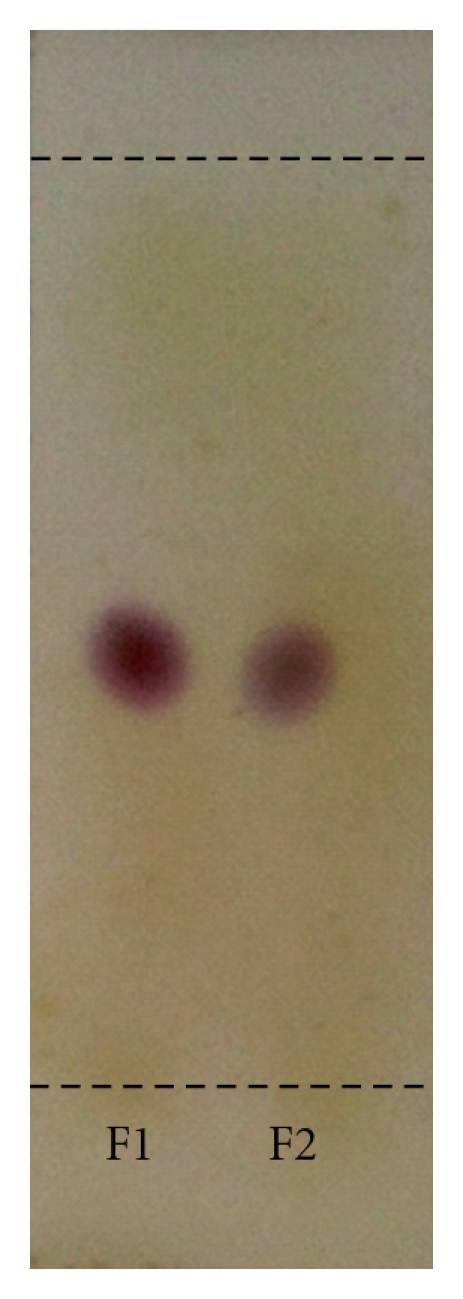
TLC of collected fractions (F1 and F2). Developing solvent: PE : EA = 3 : 1. Colorizing agent: vanillin in concentrated sulfuric acid, 5% w/v.

Capillary GC chromatograms as well as mass spectra of peaks of every collected fraction are given in Figure 2. It is indicated that the two isomers can be well separated by pGC and result in high purity products (Figure 2). Actually, the isomers are difficult to be separated by conventional method such as silica gel chromatography. As shown in Figure 3, the two separated isomers (F1 and F2) have the same retention factor (*R_f_*) values and similar color (claret-colored). Therefore, pGC shows advantages over the conventional silica gel chromatography in the separation and preparation of volatile isomers.

**Figure 4:**
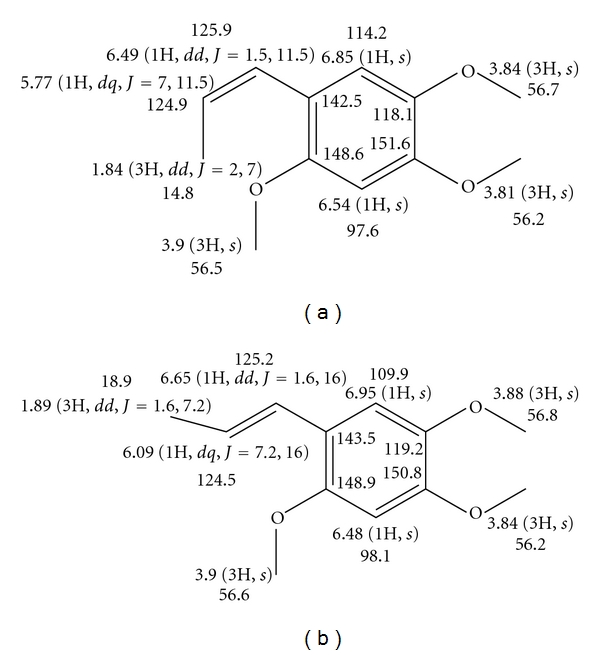
Chemical structures for *cis-*asarone (a) and *trans-*asarone (b).

Furthermore, the obtained fractions were identified by MS (Figure 2), ^1^H and ^13^C NMR spectra (shown in the appendix), and fractions 1 and 2 were identified as* cis*- and* trans*-asarone, respectively (Figure 4).

## 4. Conclusions

Preparative GC on a 3 m × 6 mm peaked column using a FID, an effluent splitter, and a fraction collector was shown with an appropriate resolution (resolution factor (*R_s_*) = 1.49) and yield to obtain pure volatile isomers at milligram level. 

## Appendix

### NMR Data of *cis*- and *trans*-Asarone: Analyzed by AV500 NMR (Bruker, Switzerland), Solvent: CDCl_3_, Internal Standard: TMS

*cis*-Asarone (F1) [35–37]
^1^H-NMR (CDCl_3_) **δ**: 6.85 (1 H, *s*, H-6), 6.54 (1 H, *s*, H-3), 6.49 (1 H, *dd*, *J *= 1.5, 11.5 Hz, H-7), 5.77 (1 H, *dq*, *J *= 7.0, 11.5 Hz, H-8), 1.84 (3 H, *dd*, *J *= 2.0, 7.0 Hz, H-9), 3.90 (3 H, *s*, 2-OCH_3_), 3.84 (3 H, *s*, 5-OCH_3_), 3.81 (3 H, *s*, 4-OCH_3_).
^13^C-NMR (CDCl_3_) **δ**: 151.6 (C-4), 148.6 (C-2), 142.5 (C-1), 125.9 (C-7), 124.9 (C-8), 118.1 (C-5), 114.2 (C-6), 97.6 (C-3), 56.7 (5-OCH_3_), 56.5 (2-OCH_3_), 56.2 (4-OCH_3_), 14.8 (C-9).

*trans*-Asarone (F2) [35, 37]
^1^H-NMR (CDCL_3_) **δ**: 6.95 (1 H, *s*, H-6), 6.48 (1 H, *s*, H-3), 6.65 (1 H, *dd*, *J *= 1.6, 16.0 Hz, H-7), 6.09 (1 H, *dq*, *J *= 7.2, 16.0 Hz, H-8), 1.89 (3 H, *dd*, *J *= 1.6, 7.2 Hz, H-9), 3.90 (3 H, *s*, 2-OCH_3_), 3.88 (3 H, *s*, 5-OCH_3_), 3.84 (3 H, *s*, 4-OCH_3_).
^13^C-NMR (CDCL_3_) **δ**: 150.8 (C-4), 148.9 (C-2), 143.5 (C-1), 125.2 (C-7), 124.5 (C-8), 119.2 (C-5), 98.1 (C-6), 56.8 (5-OCH_3_), 56.6 (2-OCH_3_), 56.2 (4-OCH_3_), 18.9 (C-9).

## References

[1] Liao WP, Chen L, Yi YH (2005). Study of antiepileptic effect of extracts from *Acorus tatarinowii* schott. *Epilepsia*.

[2] Wiseman RW, Miller EC, Miller JA, Liem A (1987). Structure-activity studies of the hepatocarcinogenicities of alkenylbenzene derivatives related to estragole and safrole on administration to preweanling male C57BL/6J x C3H/HeJ F1 mice. *Cancer Research*.

[3] Shi C, Shi YQX (2006). Research progress of asarone. *Chinese Archives of Traditional Chinese Medicine*.

[4] Limón ID, Mendieta L, Díaz A (2009). Neuroprotective effect of alpha-asarone on spatial memory and nitric oxide levels in rats injected with amyloid-*β*. *Neuroscience Letters*.

[5] Cho J, Ho Kim Y, Kong JY, Ha Yang C, Gook Park C (2002). Protection of cultured rat cortical neurons from excitotoxicity by asarone, a major essential oil component in the rhizomes of *Acorus gramineus*. *Life Sciences*.

[6] Chen YZ, Wang QW, Liang Y, Fang YQ (2007). Protective effects of beta-asarone on cultured rat cortical neurons damage induced by glutamate. *Journal of Chinese medicinal materials*.

[7] Chen YZ, Fang YQ, Wang QW, Xie YH, Kuang ZS (2007). Effects of beta asarone on morphology and cell viability in PC12 cells and cultured rat cortical neurons. *Journal of Chinese medicinal materials*.

[8] Liu J, Li C, Xing G (2010). Beta-asarone attenuates neuronal apoptosis induced by beta amyloid in rat hippocampus. *Yakugaku Zasshi*.

[9] Geng Y, Li C, Liu J (2010). Beta-asarone improves cognitive function by suppressing neuronal apoptosis in the beta-amyloid hippocampus injection rats. *Biological and Pharmaceutical Bulletin*.

[10] Zou D-J, Wang G, Liu J-C (2011). Beta-asarone attenuates beta-amyloid-induced apoptosis through the inhibition of the activation of apoptosis signal-regulating kinase 1 in SH-SY5Y cells. *Pharmazie*.

[11] Li C, Xing G, Dong M (2010). Beta-asarone protection against beta-amyloid-induced neurotoxicity in PC12 cells via JNK signaling and modulation of Bcl-2 family proteins. *European Journal of Pharmacology*.

[12] Sharma JD, Dandiya PC, Baxter RM, Kandel SI (1961). Pharmacodynamical effects of asarone and beta-asarone, naturally occurring active substances. *Nature*.

[13] Dandiya PC, Chopra YM (1970). CNS-active drugs from plant indigenous to India. *Indian Journal of Pharmacology*.

[14] Pages N, Maurois P, Delplanque B (2010). Activities of *α*-asarone in various animal seizure models and in biochemical assays might be essentially accounted for by antioxidant properties. *Neuroscience Research*.

[15] Manikandan S, Devi RS (2005). Antioxidant property of *α*-asarone against noise-stress-induced changes in different regions of rat brain. *Pharmacological Research*.

[16] Pérez-Pastén R, García RV, Garduño L (2006). Hypolipidaemic and antiplatelet activity of phenoxyacetic acid derivatives related to *α*-asarone. *Journal of Pharmacy and Pharmacology*.

[17] Labarrios F, Garduño L, Vidal MDR (1999). Synthesis and hypolipidaemic evaluation of a series of *α*-asarone analogues related to clofibrate in mice. *Journal of Pharmacy and Pharmacology*.

[18] Garduño L, Salazar M, Salazar S (1997). Hypolipidaemic activity of *α*-asarone in mice. *Journal of Ethnopharmacology*.

[19] Rodríguez-Páez L, Juárez-Sanchez M, Antúnez-Solís J, Baeza I, Wong C (2003). *α*-asarone inhibits HMG-CoA reductase, lowers serum LDL-cholesterol levels and reduces biliary CSI in hypercholesterolemic rats. *Phytomedicine*.

[20] Argüelles N, Sánchez-Sandoval E, Mendieta A (2010). Design, synthesis, and docking of highly hypolipidemic agents: schizosaccharomyces pombe as a new model for evaluating *α*-asarone-based HMG-CoA reductase inhibitors. *Bioorganic and Medicinal Chemistry*.

[21] Sandeep D, Nair CKK (2011). Radioprotection by *α*-asarone: prevention of genotoxicity and hematopoietic injury in mammalian organism. *Mutation Research*.

[22] Schmidt GH, Streloke M (1994). The essential oil of Indian *Acorus calamus* (L.) shows potential for stored-product pest control. Its active ingredient, *β*-asarone, has toxic and sterilizing effects. *Journal of Stored Products Research*.

[23] Yang FQ, Li SP, Zhao J, Lao SC, Wang YT (2007). Optimization of GC-MS conditions based on resolution and stability of analytes for simultaneous determination of nine sesquiterpenoids in three species of *Curcuma* rhizomes. *Journal of Pharmaceutical and Biomedical Analysis*.

[24] Yang FQ, Li SP, Chen Y (2005). Identification and quantitation of eleven sesquiterpenes in three species of *Curcuma* rhizomes by pressurized liquid extraction and gas chromatography-mass spectrometry. *Journal of Pharmaceutical and Biomedical Analysis*.

[25] Ke XH, Wei G, Fang YG (2002). Assay of *β*-asarone and *α*-asarone in Rhizoma acori tatarinowii by HPLC. *Chinese Traditional Patent Medicine*.

[26] Zhang HZ, Liu J, Di ZZ, Guo ZW (2009). Determination of *α*-asarone in different Radix et Rhizoma Asari by HPLC. *Herald of Medicine*.

[27] Li YM, Sui DS (2009). Identification of Rhizoma Acori Talarinowii and determination of its *β*-asarone content. *Traditional Chinese Drug Research & Clinical Pharmacology*.

[28] Dong Y, Shi RB, Liu B (2007). Quantitative determination of *α*-asarone and *β*-asarone in effective fraction of *Acorus tatarinowii* Schott. *Journal of Beijing University of Traditional Chinese Medicine*.

[29] Yu SM, Kim EK, Lee JH, Lee KR, Hong J (2011). Development of fingerprints for quality control of *Acorus* species by gas chromatography/mass spectrometry. *Bulletin of the Korean Chemical Society*.

[30] Wei G, Fang YQ, Liu DH, Lin SF (2004). Study on GC-MS fingerprint analysis in rhizome of volatile oil of *Acorus tatarinowii*. *China journal of Chinese materia medica*.

[31] Zeng Z, Ye ZN, Shen MT, Zhang T, Zhang YQ, Fu L (2011). Study on the volatile constituents of *Acorus tatarinowii* Schott from different growing areas. *Journal of Instrumental Analysis*.

[32] Liu CH, Liu XJ, Yang HS (2006). GC-MS analysis of essential oils from *Acorus tatarinowii* Schott. *Chinese Archives of Traditional Chinese Medicine*.

[33] Oprean R, Tamas M, Roman L (1998). Comparison of GC-MS and TLC techniques for asarone isomers determination. *Journal of Pharmaceutical and Biomedical Analysis*.

[34] Yang FQ, Wang HK, Chen H, Chen JD, Xia ZN (2011). Fractionation of volatile constituents from curcuma rhizome by preparative gas chromatography. *Journal of Automated Methods and Management in Chemistry*.

[35] Patra A, Mitra AK (1981). Constituents of *Acorus calamus*: structure of acoramone. Carbon-13 NMR spectra of *cis*- and *trans*-asarone. *Journal of Natural Products*.

[36] Zhu MJ, Tan NH, Ji CJ, Xu JJ, He WJ, Zhang YM (2010). Chemical constituents from petroleum ether fraction of ethanol extract of *Acorus tatarinowii*. *China Journal of Chinese Materia Medica*.

[37] Xu JH, Lu QH, Zhao Y (2007). Studies on chemical constituents of green algae *Ulva pertusa*. *Zhongguo Zhongyao Zazhi*.

